# How Are Adenosine and Adenosine A_2A_ Receptors Involved in the Pathophysiology of Amyotrophic Lateral Sclerosis?

**DOI:** 10.3390/biomedicines9081027

**Published:** 2021-08-17

**Authors:** Akihisa Mori, Brittany Cross, Shinichi Uchida, Jill Kerrick Walker, Robert Ristuccia

**Affiliations:** 1 Kyowa Kirin Co., Ltd., Otemachi, Chiyoda-ku, Tokyo 100-0004, Japan; shinichi.uchida.fd@kyowakirin.com; 2 Kyowa Kirin, Inc., Bedminster, NJ 07921, USA; brittany.cross.b2@kyowakirin.com (B.C.); jill.kerrickwalker.bb@kyowakirin.com (J.K.W.); Robert.Ristuccia.bt@kyowakirin.com (R.R.)

**Keywords:** adenosine, adenosine A_2A_ receptor, amyotrophic lateral sclerosis

## Abstract

Adenosine is extensively distributed in the central and peripheral nervous systems, where it plays a key role as a neuromodulator. It has long been implicated in the pathogenesis of progressive neurogenerative disorders such as Parkinson’s disease, and there is now growing interest in its role in amyotrophic lateral sclerosis (ALS). The motor neurons affected in ALS are responsive to adenosine receptor function, and there is accumulating evidence for beneficial effects of adenosine A_2A_ receptor antagonism. In this article, we focus on recent evidence from ALS clinical pathology and animal models that support dynamism of the adenosinergic system (including changes in adenosine levels and receptor changes) in ALS. We review the possible mechanisms of chronic neurodegeneration via the adenosinergic system, potential biomarkers and the acute symptomatic pharmacology, including respiratory motor neuron control, of A_2A_ receptor antagonism to explore the potential of the A_2A_ receptor as target for ALS therapy.

## 1. Introduction

Amyotrophic lateral sclerosis (ALS), sometimes known as Lou Gehrig’s disease, is a fatal neurodegenerative disease characterized by progressive muscular paralysis reflecting degeneration of pyramidal motor neurons in the primary motor cortex, corticospinal tracts, brainstem and spinal cord [1]. During ALS progression, both the upper (cortical) motor neurons and the lower (spinal cord) motor neurons degenerate, causing a progressive and terminal atrophy of skeletal muscles. All muscles under voluntary control are affected, and individuals with ALS progressively lose their strength and their ability to move. Once the diaphragm and the muscles in the chest wall fail, people lose the ability to breathe without ventilation support [1]. Globally, the average age of onset of ALS is currently 58–60 years, and the average survival from onset to death is 3–4 years [2]. Approximately 90–95% of all ALS cases are of unknown etiology and are referred to as ‘sporadic’ or ‘idiopathic’ ALS [2,3]. Most other ALS cases are familial, with a Mendelian pattern of inheritance resulting from a number of gene mutations, including mutations in the genes for superoxide dismutase 1 (SOD1), TAR DNA-binding protein 43 (TDP-43), fused in sarcoma (FUS) and C9orf72 [4].

ALS is a complex, multifactorial and multi-system disease, for which the full pathophysiological mechanisms of degeneration remain unclear. Known mechanisms include RNA dysfunction, protein misfolding and aggregation, mitochondrial dysfunction, neuroinflammation, neuromuscular junction abnormalities, immune system deficiency, cytoskeletal aberrations, growth factor dysfunction, oxidative stress, axonal disruption and apoptosis, excitotoxicity, activation of nucleases and proteases, and abnormal calcium metabolism [4,5,6]. Similar to their now generally well-accepted staging theory in Parkinson’s disease (PD) [7], Braak and colleagues have suggested ALS could be a model of corticofugal axonal spread, where motor neuron degeneration initially results from failure of enzymatic machinery at the level of the cell body and proximal parts of the axon that then propagates in a corticofugal way due to impaired axonal transport [8]. Others have argued that ALS starts with nerve terminal dysfunction, with consequent synaptic dysfunction and then progressing in a ‘dying back’ process [9]. These hypotheses are not mutually exclusive [10].

Regardless of mechanisms, it is increasingly apparent that ALS involves different cell types (including interneurons, astrocytes, microglia, Schwann cells, skeletal muscle cells and oligodendrocytes), the communication between them, and that the degeneration of each cell population significantly contributes to the relentless progression of the disease [11]. Like other neurodegenerative disorders, a major problem for developing treatments is by the time patients are diagnosed, they have already had significant motor neuron degeneration. It has been suggested that early intervention focusing on motor neuron terminals could potentially delay or prevent the progression of the disease. Accumulating evidence suggests an early dysfunction of the adenosinergic system in ALS. Adenosine is a ubiquitous neurochemical, modulating synaptic transmission at pre-, post- and non-synaptic levels and is involved in several essential actions. In this article, we review the possible mechanisms of chronic neurodegeneration via the adenosinergic system, the potential of uric acid as a biomarker and the acute symptomatic pharmacology (including phrenic motor facilitation) of A_2A_ receptor antagonism to explore the potential of the A_2A_ receptor (A_2A_R) as target for ALS therapy.

## 2. Adenosine as a Neuromodulator

**Figure 1:**
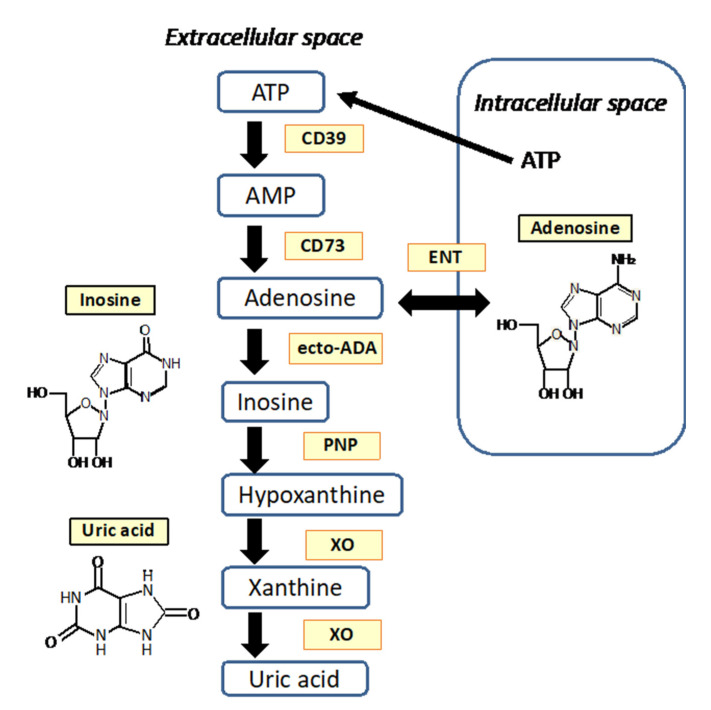
Extracellular adenosine production and metabolism. ATP: adenosine triphosphate; AMP: adenosine monophosphate; CD39: triphosphate diphophahydrolase-1; CD73: ecto-5′ nucleotidase; ecto-ADA: adenosine ecto-deaminase; ENT: equilibrative nucleotide transporter; PNP: purine nucleoside phosphorylase; XO: xanthine oxidase [19,20].

Adenosine is a neuromodulator produced both intracellularly as well as in the extracellular space. Intracellular production occurs via metabolic pathways that are highly regulated and include adenosine triphosphate (ATP) production via adenosine monophosphate (AMP) by adenosine kinase, nucleotide/DNA synthesis and the S-adenosylhomocysteine pathway [12]. Once produced inside a cell, adenosine can be transported into the extracellular space via the equilibrative nucleotide transporters ENT1 and ENT2. Located on most cells, these transporters enable bidirectional transport across the cell membrane and ensure there is always a finite amount of adenosine in the extracellular space [13,14]. Adenosine is also produced in the extracellular space through the metabolism of ATP via ectonucleotidases. In the first step of this process, ATP is converted into AMP by triphosphate diphosphohydrolase-1 (CD39). AMP is then converted into adenosine by ecto-5′nucleotidase (CD73) [12,15] (Figure 1). Importantly, extracellular adenosine concentrations can originate from both neurons and glia [16]. Adenosine does not function as a central neurotransmitter in the traditional sense. Rather, it is produced as a result of cellular metabolism and transported across the cell membrane or within the extracellular space such that adenosine is always present in the extracellular space. Extracellular adenosine levels increase as neuronal activity increases, and thus the adenosinergic system provides a level of neuronal homeostatic control [17,18].

Several mechanisms for activity-dependent increases in extracellular adenosine level have been proposed because adenosine production is likely to vary by brain region. Using CD73 knockout (KO) mice, Klyusch et al. showed two parallel pathways of central adenosine release: one that is indirect via glutamate receptor-dependent release of ATP and a second of equal amplitude that has no dependence on prior release of ATP and thus represents the direct release of adenosine [21]. This component of adenosine release was modulated by metabotropic glutamate (mGlu4) receptor activation, strongly supporting adenosine release by exocytosis from parallel fibers of the cerebellum. Similarly, Pajski et al. showed adenosine production can be triggered by nerve stimulation (action potential-dependent) mechanism in striatal brain tissues, and both low- and high-frequency stimulated release were almost completely blocked by removal of calcium, indicating activity dependence [22]. Reducing dopamine efflux did not affect adenosine release, but inhibiting ionotropic glutamate receptors did, supporting the idea that striatal adenosine release may be affected by downstream effects of glutamate [22]. In spinal cord slices of the dorsal horn, it has been reported that adenosine seems to be available by the breakdown of AMP in the extracellular space where both prostatic acid phosphatase (PAP) and CD73 have been implicated [23,24]. Genetic deletion of both ectonucleotidases in double knock-out mice reduced, but did not eliminate, the production of adenosine from extracellular AMP, suggesting at least one additional AMP ectonucleotidase was present in dorsal root ganglia (DRG) neurons and spinal cord [23], and further study found tissue-nonspecific alkaline phosphatase (TNAP) can dephosphorylate AMP in these tissues. TNAP is also widely expressed in the brain, suggesting a role for this enzyme in the CNS [25], where it also hydrolyzes extracellular ATP to promote the axonal growth of hippocampal neurons [26] and can serve as a source of extracellular adenosine in the hippocampus when CD73 is deleted [27].

Considering the regional differences in spontaneous, transient adenosine release [28], it appears there may still be a few, as yet unknown, mechanisms that act simultaneously to form extracellular action potential-stimulated adenosine, which depend upon each individual brain area. For example, using brain slices from different areas, Lee and Vento reported that the frequency of adenosine release is highest in the prefrontal cortex, while the hippocampus has the largest concentration, and the thalamus has the longest duration of release [28]. More work is needed to understand the availability of adenosine to work as a neuromodulator in the different brain regions, and it is important to acknowledge that current understanding is limited as most studies have been done using in vitro brain slices, not in vivo models.

## 3. Adenosine Receptors

To date, four subtypes of adenosine receptor have been identified in mammalians, A_1_, A_2A_, A_2B_, and A_3_, all of which are G-protein coupled receptors (Table 1). A_1_ receptors are expressed on both pre- and postsynaptic sites and are coupled to pertussis toxin-sensitive Gαi and Gαo. Binding of adenosine to A_1_ receptors leads to inhibition of adenylyl cyclase (AC) and downstream reduction in cAMP-dependent kinase (PKA) [29,30]. A_1_ receptor stimulation also activates phospholipase C (PLC) [31]. This has the effect of modulating the release of neurotransmitters and neuropeptides from neurons [29,30]. Like A_1_ receptors, A_2A_Rs signal through the AC-cAMP-PKA pathway [12]. This leads to activation of downstream targets, such as cAMP response element-binding protein (CREB), that promote transcription of genes related to cell survival and neuronal plasticity [32,33].

The distribution of adenosine receptors varies, with the A_1_, A_2B_, and A_3_ subtypes widely expressed throughout the central nervous system (CNS) and peripheral organs/tissues (albeit at relatively low densities for A_2B_ and A_3_ receptors) [34,35]. A_2A_Rs, on the other hand, have a relatively limited distribution, with expression in CNS, especially highly restricted to the striatum, external globus pallidus, nucleus accumbens, and olfactory tubercle [17,18]. In the peripheral organs/tissues, A_2A_Rs have been identified on a few organs, blood vessels, immune cells, platelets and microglia [34,35].

**Table 1:** Adenosine receptor localization.

Adenosine Receptor Subtype	Central Nervous System	Peripheral Organs/Tissues and Non-neuronal Cells
A_1_	Widely distributed with highest levels in the cerebral cortex, hippocampus, cerebellum, thalamus, brain stem and dorsal horn of the spinal cord [35,36,37,38]	Widely distributed, including mononuclear cells in the blood, heart, kidney, adipose tissue [34,35,39,40]
A_2A_	Highly concentrated in dorsal and ventral striatum (on striatopallidal medium spiny neurons (MSNs)). Additionally expressed in the globus pallidus (external), nucleus accumbens, olfactory tubercle [17,18,41]. Expressed in lower levels in the hippocampus, thalamus, cerebellum, cerebral cortex [38,42,43,44] and spinal cord motor neurons [36,41,45].	Spleen, thymus, blood vessels, heart, lung, immune cells, platelets, glial cells [34,35,46]
A_2B_	Widely distributed (low density) [34,35]	Widely distributed (very low density). Higher levels in the cecum, colon, bladder, macrophages, mast cells [34,35]
A_3_	Widely distributed (low density) [34,35]	Widely distributed (low density). Higher levels in mast cells, eosinophils [34,35,47]

Since it was first cloned in the late 1980s [48], the A_2A_R has been of particular interest in movement disorders such as PD because of their selective expression in the brain regions involved in regulating motor control (i.e., the basal ganglia) and the pathogenesis of symptomatic motor dysfunction [42]. The adenosine A_2A_R antagonist istradefylline (formerly known as KW-6002) is the first adenosinergic antiparkinsonian agent to be approved (Japan and USA) as a symptomatic treatment for PD [49]. The journey through research and development provides a good example of translational research, where the evidence base was carefully constructed according to the following:

- Identification of A_2A_R-specific expression in the medium spiny neurons (MSNs, also known as spiny projection neurons), projecting through the striatum to GPe [50].
- Synthesis and identification of selective A_2A_R antagonists [51,52].
- Demonstration of A_2A_ antagonist efficacy in functional animal models for PD [53].
- Discovery of physiological significance of A_2A_Rs in the MSN and establishing the mechanism of action for A_2A_R antagonism in PD therapy [43].
- Pathophysiological change with increased level of A_2A_Rs in progression of PD [54,55,56,57,58].
- Proof-of-concept clinical studies in PD patients, translating A_2A_R antagonist pharmacology into clinical manifestation [49,59].
- Clinical development for regulatory registration [49,60].

This translational process may provide a template pathway for investigations of adenosinergic therapeutics for ALS. The analogies between the two neurodegenerative diseases are interesting, not least because of the evidence for adenosinergic system involvement in ALS, as well as the evidence for A_2A_R expression in spinal cord motor neurons [36,41].

## 4. Pathophysiology in Adenosine Levels and A_2A_ Receptor Density in ALS

Several studies have suggested that adenosinergic function (i.e., adenosine levels, adenosine receptors) within different tissues/areas in the central nervous systems seems to be enhanced as ALS progresses. Evidence for increased adenosine levels in the cerebrospinal fluid of patients with ALS (*n* = 12) [61] was already available in the late 1990s, but it has only recently been demonstrated that the extracellular adenosine concentration, which can be increased by loss of astrocyte adenosine deaminase (ADA), is critical to induce motor neuron toxicity in ALS [62]. In C9orf72 cells and astrocytes derived from sporadic ALS patients, the metabolism of inosine was shown to be reduced as a result of the reduced activity of ADA. ALS induced astrocytes were more susceptible to adenosine induced cell loss than control induced astrocytes and were protected by inosine supplementation, resulting in an increase in motor neuron survival in co-culture with induced astrocytes. This suggests that adenosine levels are, at least in part, a cause (and not just a consequence) of the progressive motor neuron loss in ALS.

Since the early receptor binding studies first suggested expression of adenosine receptors in the spinal cord [36], it has been directly observed that adenosine A_2A_Rs are highly enriched in non-astroglial cells, including motor neurons in the spinal cord ventral horns, compared to levels in the cortex and hippocampus. In contrast, levels of adenosine A_1_ receptors in the spinal cord are lower than in other areas. Interestingly, a clear trend to the upregulation of A_2A_, but not A_1_, receptors has been found in samples from post-mortem patients with ALS [63]. Additionally, studies using the symptomatic SOD1^G93A^ mouse model of ALS have reported that A_2A_R expression in SOD1^G93A^ mice spinal cords is increased 3-fold compared to wild-type mice, with no significant changes in A_1_ receptor expression, in the early symptomatic (symptomatic onset) phase [64]. Conversely, symptomatic SOD1^G93A^ mice have been shown to have a dramatic decrease in A_2A_Rs in the spinal cord [65]. Both these observations suggest alteration of A_2A_R expression during ALS progression is related to the SOD1 mutation.

Enhanced A_2A_ (but not A_1_) receptor expression and signaling has also been detected in non-motor areas (i.e., hippocampus) of pre-symptomatic SOD1 mutation mice [66]. Rei et al. [66] have further shown that, while blockade of A_2A_Rs with istradefylline did not alter the receptor levels in wild-type mice, chronic treatment normalized A_2A_R expression in SOD1^G93A^ mice down to wild-type levels. It seems unlikely that a receptor antagonist would induce down regulation; however, this requires confirmation.

Interesting analogies in pathophysiological changes of A_2A_Rs during disease progression can be made between ALS and other neurodegenerative diseases. In patients with PD, increased striatal and pallidal (GPe) A_2A_R density has been demonstrated, both in postmortem brain tissue [54,55] and using PET imaging [56,57,58]. Further work has also shown increased putaminal density in the early ‘pre-symptomatic’ phase of PD (Braak PD stages of 1–2) [55] as well as significant changes in receptor expression during more advanced PD when patients were experiencing motor complications [54,56,57,58]. This localization is not just interesting due to its discrete nature but also due to the functional significance of the areas linking A_2A_ expression and PD. Postmortem evaluation of the cortex of patients with frontotemporal lobe dementia (FTLD) has also demonstrated an increase in A_2A_Rs of the temporal cortex [67]. The study also demonstrated an association between the increase in A_2A_Rs and phosphorylated tau protein, suggesting a sequential process resulting in cognitive impairment [67]. Like these diseases, the exact timing and conditions for A_2A_R changes during ALS progression remain to be investigated.

## 5. Pharmacology of Adenosine A_2A_ Receptor Blockade on ALS Animal Models

Despite the growing body of evidence for increased adenosine levels and upregulation of A_2A_R levels in human ALS and ALS models (Table 2), pharmacological outcomes, using both A_2A_ agonists/antagonists, vary considerably (Table 3).

In SOD1^G93A^ mice, A_2A_ antagonism with istradefylline has demonstrated beneficial effects, including motor neuroprotection. Ng et al. [64] demonstrated adenosine treatment induced embryonic stem cell-derived motor neuron (ESMN) cell death in cultures, while application of istradefylline significantly protected against death of EMSNs co-cultured with SOD1^G93A^ + astrocytes. From a motor function perspective, daily treatment of the A_2A_ antagonist and partial genetic ablation of the A_2A_R significantly delayed disease progression in SOD1^G93A^ mice, which was evaluated by longitudinal grip strength change [64]. Istradefylline has also been shown to protect against kainate-induced motor neuron death as well as time-dependent death of motor neurons by expression of mutant forms of SOD1 and mutant p150^glued^ subunit of dynactin in rat spinal cord cultures. This study has found that istradefylline led to a substantial reduction in phosphor Trk, suggesting A_2A_ antagonism inhibits activation of the receptor tyrosine kinase (Trk) and downstream signaling of Trk-B, which co-localizes with the A_2A_R in motor neurons [70].

Rei et al. [66] have found, using in vitro hippocampal slices from SOD1^G93A^ mice, that, in comparison with those from wild-type mice, glutamatergic pre-synaptic function was enhanced with up-regulated A_2A_Rs in pre-symptomatic mice. By contrast, in symptomatic mice, NMDA glutamatergic transmission and its plasticity (i.e., long-term potentiation (LTP)) were impaired but were rescued by A_2A_R blockade. Since the study was done in a non-motor brain area, it is less conclusive if this sequential change, from the pre-symptomatic to the symptomatic phase of ALS, can be translated to progressive motor dysfunction in ALS. However, these findings may contribute to further understanding a mechanism for non-motor symptoms of ALS, such as cognitive dysfunction. Similar changes of A_2A_R neuronal plasticity are also reported in corticostriatal glutamatergic long-term depression (LTD) using in vitro slices from DYT1 dystonia model mice. In the symptomatic disease state, LTD was impaired, which could be recovered by A_2A_R blockade resulting in motor improvement [79].

Electrophysiological study of phrenic-nerve hemidiaphragm prepared from in pre-symptomatic SOD1 mutation mice (4–6 weeks old) has also demonstrated that the selective A_2A_R agonist, CGS 21680, significantly enhanced neuromuscular junction (NMJ) transmission, the effect being of higher magnitude than age-matched control littermates [75]. However, in the preparation from symptomatic phase mice (12–14 weeks old), the A_2A_R-mediated effects disappear (although NMJ transmission from wild-type mice was increased by A_2A_R stimulation) [75]. These pathophysiological findings may suggest that A_2A_R function and/or sensitivity alters between pre-symptomatic to symptomatic phases in ALS, which is in line with A_2A_R expression change in SOD1^G93A^ mice, as mentioned previously. This A_2A_R-mediated NMJ control in the phrenic nerve is also key for symptomatic therapeutic strategies in ALS (see Section 6 below). Adenosine A_1_ receptor activation, using the same pre-symptomatic phase preparation, decreased NMJ transmission but, during the symptomatic phase, increased its tonic activation [77]. Taken together with A_2A_ changes, this suggests physiological interactions between excitatory A_2A_ and inhibitory A_1_ receptors [80] are disrupted during presynaptic regulation, leading to a higher level of adenosine than that in age-matched controls [77].

There are conflicting data regarding the pharmacology of A_2A_Rs in SOD1 mutation mice. In contrast to previous discussion, it has been reported that, in an in vivo study using presymptomatic SOD1^G93A^ mice (starting at 8 weeks and continued until 12 weeks), the A_2A_ agonist CGS21680 slowed the onset of motor neuron degeneration with muscle weakness. This was considered due to an A_2A_R-mediated activation of brain-derived neurotrophic factor (BDNF) truncated receptor (TrkB) signaling independent of neurotrophines [74]. However, a further in vivo study with presymptomatic SOD1 mutation mice showed that neither the stimulation nor blockade of A_2A_Rs by CGS21680 (i.p) or istradefylline via drinking water, respectively, modified the progressive loss of motor skills or survival of the mice [76]. Although the route of administration for the drug has been suggested to be the root cause of the conflicting data [81], the effect of A_2A_R agonism in the presymptomatic phase needs to be further investigated.

In another SOD1^G93A^ mouse study, caffeine intake was reported to shorten survival. While the authors at the time considered this an “unexpected result”, there are two possibilities that may explain the observation [65]. Since caffeine is a non-selective adenosine antagonist exerting various pharmacological effects, it may be that the decreased survival is not attributable to A_2A_R antagonism but other receptor characteristics of the drug. Another possible explanation may be reduced A_2A_R-induced neurotrophic support since the A_2A_R is closely involved in the regulation of vascular endothelial growth factor (VEGF) expression [82]. On the other hand, the authors also found a dramatic down-regulation of spinal cord A_2A_Rs, making it hard to speculate that the effects were mediated by A_2A_R inhibition [65]. Moreover, the outcome of a recent pooled analysis of clinical cohort studies in patients with ALS did not support associations of ALS mortality risk with caffeine consumption [83].

TAR DNA binding protein (TDP-43 transgenic) transgenic mice are used as another ALS model because cytoplasmic mislocalization of TDP-43 from the nucleus is considered a hallmark of early event for the pathogenesis of ALS [84]. However, only a few studies using this model have been done to investigate the contribution of adenosine receptors. Liu et al. [72] suggested that elevated oxidative stress might cause the abnormal activation of AMPK, subsequently causing the mislocalization of TDP-43. Using the A_2A_ agonist JMF1907 to suppress AMPK activity via cAMP stimulation, they demonstrated that (i) activation of A2AR rescues the AMPK-triggered mislocalization of TDP-43 in a motor neuron cell line (NSC34), which was blocked by the A_2A_ antagonist SCH21680, and (ii) treatment with JMF1907 improved motor function in rotarod performance and forelimb grip strength [72]. Finally, JMF1907 also inhibits the adenosine transporter ENT1 [85], causing an increase in adenosine levels. Thus, in contrast to SOD1 mutation models, the TDP-43-related ALS model can demonstrate therapeutic effects of A_2A_R stimulation. However, AMPK is activated in the spinal cord of SOD1^G93A^ mice at disease onset [86,87], but it is suppressed in transgenic TDP-43 A315T mice at the presymptomatic and symptomatic stages [87], making regulation of AMPK during disease progression a priority area for further investigation.

In summary, the potential contribution of A_2A_R pharmacology in ALS can be considered to depend on two factors:

- Disease stage (pre-symptomatic phase, onset of symptomatic phase and end stage);
- The ALS model used and the mechanisms underlying the motor neuron disease.

## 6. Uric Acid as a Proposed Biomarker in Patients with ALS

Adenosine in neuronal systems follows a well-recognized metabolic pathway (Figure 1) breaking down into inosine through the activity ADA [88] with the clearance mediated via nonconcentrating nucleoside transporters. It has been suggested that, whereas neurons are enriched in adenosine kinase, ADA is more abundant in astrocytes [62,88]. The end metabolite in humans, uric acid (UA), is well known to have antioxidant properties [89,90,91].

Serum UA has been proposed to be a biomarker of ALS progression (especially in the early phases) [92], and there is accumulating evidence demonstrating that serum UA levels correlate with ALS progression as measured by the ALS Functional Rating Scale-Revised (ALSFRS-R) [93,94]. Meta-analyses also support an inverse association of serum UA levels with risk of death among ALS patients [95]. Other studies have shown a significant survival advantage of higher UA levels in male, but not female, patients [96]. Again, there is an interesting analogy to be made with other neurodegenerative disorders since UA levels are also found to be inversely associated with the risk of PD and Alzheimer’s disease (AD) [97,98,99,100]. In addition, studies in Huntington’s disease, multiple system atrophy and mild cognitive impairment have also demonstrated a correlation between higher UA levels and slower clinical progression [101,102,103,104].

Much of the current literature postulates that UA plays an important role in ameliorating oxidative stress, and research has focused on addressing UA-induced neuroprotective effects. Authors often suggest that UA produced from inosine via xanthine [105] may provide some level of neuroprotection, partly based on an antioxidant action [106,107], since oxidative stress is thought to induce motor neuron death and promote the pathogenesis of ALS [108,109,110]. Additionally, UA-induced protection of spinal cord neurons from glutamatergic excitotoxicity via astrocytes has also been proposed as another possible mechanism [111]. However, a study by Allen et al., using inosine supplements that significantly reduced the induced astrocyte-mediated toxicity toward motor neurons, found that increased UA levels from inosine were not always correlated with motor neuron survival increases. This led them to suggest that the protection they observed was not via UA production but another pathway triggered by inosine (i.e., lactate production induced by the increased glycolytic capacity) [62]. Thus, an alternative or additional thought may be that serum UA is actually a marker of remaining extracellular adenosine levels in ALS. This may suggest lower serum UA levels indicate higher levels of adenosine in neuronal systems, including motor neurons, which may increase risk for ALS induction and/or progression in particular neuronal systems. However, whether lower serum UA is attributable to reduction in adenosine metabolism (i.e., suppression of ADA) is yet to be investigated.

Ectonucleotidase-mediated ATP catabolism (CD73-mediated adenosine formation) provides a powerful mechanism to control the levels of extracellular adenosine. Orr et al. have shown that the conversion of ATP to adenosine by activated microglia leads to activation of the adenosine A_2A_R and consequent microglial process retraction into an amoeboid shape (considered a hallmark of neuroinflammation or trauma) [112]. By connecting the neurodegenerative processes and mechanisms related to both increased adenosine levels and adenosine A_2A_R activation, Meng et al. have recently suggested that CD73 provides a self-regulating feed-forward adenosine formation to activate striatal A_2A_Rs in cells that release pro-inflammatory cytokines causing neurodegeneration [113]. Using the 1-methyl-4-phenyl-1,2,3,6-tetrahydropyridine (MPTP) model of PD, they showed that limiting CD73-derived adenosine substantially suppressed microglia-mediated neuroinflammation. Based on experiments using CD73 KO mice, they further showed CD73 inactivation suppressed A_2A_R induction and A_2A_R-mediated pro-inflammatory responses [113].

In other neurodegenerative diseases such as Alzheimer’s disease and frontotemporal degeneration, increased astrocytic A_2A_R expression is also correlated with memory deficits [67,114], and studies in a mouse model of tauopathy have shown that A_2A_Rs exacerbate tau phosphorylation and memory loss [67]. A_2A_Rs are also known to be upregulated in stroke [115], and studies have shown selective A_2A_R antagonism reduces ischemic brain damage and neurological deficit [116,117] via mechanisms including inhibition of oligodendrocyte-mediated neuroinflammation [118]. For ALS patients, increased A_2A_R expression (versus healthy controls) has also been demonstrated in lymphocytes, and the density correlated with ALSFR-R scores [68]. In addition, the lymphocytes from patients with ALS had a higher potency for A_2A_R functional activation, represented by cAMP levels, than those from healthy subjects.

These lines of converging evidence seem to suggest that A_2A_R changes in non-neuronal cells may be a reliable indicator for what happens in A_2A_R-induced neurodegeneration and could be a key process for neuronal degeneration triggered via non-neuronal cells. In the PD model, CD73 activation was induced by the neurotoxin MPTP, which causes dopaminergic degeneration [113], and this approach could be adapted for ALS by developing a model of ADA deficiency. Interestingly, in mouse models of spinal cord injury, CD73 expression was also upregulated in microglia. The authors of the study concluded CD73 has an anti-inflammatory role, attributed to inhibition of macrophages/microglia polarization [119]. Thus, CD73 in microglia may be a specific target to be investigated to unravel the entire process from cause to consequence in the pathogenesis of ALS. Other potentially translatable mechanisms of adenosine A_2A_R-mediated neurotoxicity in PD have been described and are extensively reviewed by Chen and Schwarzschild [120].

While it can now be assumed that an increase in extracellular adenosine levels due to loss of ADA and upregulation of A_2A_Rs contributes to motor neuron death and functional impairment in ALS, several questions remain:

- What is the sequence for initiating motor neuron death? Increased level of adenosine and/or via adenosine A_2A_R activation?
- What causes loss of ADA?
- What are the mechanisms that drive the upregulation/down regulation of A_2A_Rs?
- What are the timings by which the alterations of the adenosinergic system occur during ALS pathogenesis in patients and animal models?
- What are the processes of each event in the entire pathogenesis of ALS from pre-symptomatic, symptomatic and end stages?
- How can plasma UA be utilized as a biomarker for diagnosis and therapy?
- Can A_2A_R antagonists (and agonists) be useful pharmacotherapy during an ALS patient’s journey, and if so, what is the optimal timing for such therapy?

## 7. A_2A_ Receptors in Respiratory Motor Neurons and tAIH Treatment for ALS

The respiratory neuronal network must continuously adjust due to the dynamic demands throughout one’s life to maintain homeostasis, including adjustments for disease onset. These regulatory strategies are achieved through various feedback, feedforward, and adaptive mechanisms. Several clinical disorders challenge the neuronal control of respiratory motor output, including neuromuscular disorders such as spinal cord injury (SCI) and ALS [121,122,123,124]. In fact, a major cause of mortality in both SCI and patients with ALS is the disruption and degeneration of the respiratory motor neurons [122,125]. Eventually, a diminished ability to generate spinal respiratory motor nerve activity exceeds the compensation capacities of the respiratory system and compromises breathing, leading to respiratory failure [122,125,126,127]. In the case of ALS, neuronal loss eventually leads to ventilator dependence or death [122,126,127]. Therefore, it is critical to develop new strategies that restore neuronal motor activity and preserve independent breathing in these patients.

**Figure 2:**
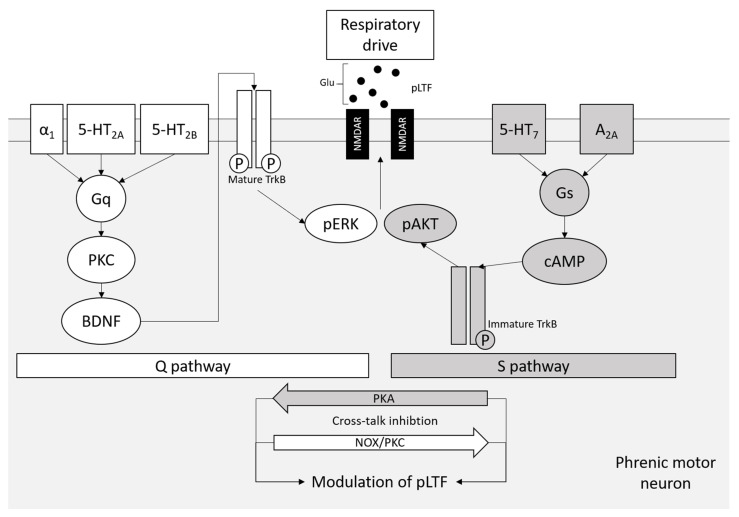
The Q and S pathways to long-lasting phrenic motor facilitation. BDNF: brain-derived neurotrophic factor; ERK: extracellular signal-related protein kinase; Glu: glutamate; Gq: Gq protein-coupled receptor; Gs: Gs protein-coupled receptor; NOX: nicotinamide adenine dinucleotide phosphate (NADPH) oxidase; pLTF: phrenic long-term facilitation; pMF: phrenic motor facilitation; PKA: protein kinase A; PKC: protein kinase C; TrkB: tropomyosin-related kinase B.

When perturbations occur, one of the breathing control strategies for the neuronal respiratory system is plasticity [123,128,129,130,131]. Phrenic motor facilitation (pMF) is a form of motor plasticity induced by neuromodulators, such as serotonin and adenosine, to increase the neural output of the phrenic nerves [123,124,132,133,134]. A specific form of pMF, known as phrenic long-term facilitation (pLTF), is excited when exposed to acute intermittent hypoxia (AIH) and leads to a long-lasting increase in phrenic motor output [128,135,136,137,138,139]. pLTF is pattern-sensitive as it requires intermediate hypoxia rather than continuous hypoxia [140]. AIH-induced pLTF is also pattern-sensitive to the severity of hypoxia via two distinct cellular pathways [133,140] (Figure 2).

Moderate AIH (mAIH) initiates pLTF via the Q pathway to pMF activity. It is called the Q pathway because it is induced by spinal Gq protein-coupled serotonin 2 (5-HT_2_) metabotropic receptors [128,137,141,142,143,144]. The Q pathway also requires downstream extracellular signal-related protein kinase (ERK) mitogen extracellular kinase (MEK) activity and new protein synthesis of BDNF [145,146]. New BDNF synthesis leads to activation of its receptor, tropomyosin-related kinase B (TrkB), and protein kinase C theta (PKCθ) activity [145,147,148,149,150]. When AIH is severe (sAIH), pLTF activation’s dominant mechanism is through the S pathway to pMF activity [128,151]. The S pathway is initiated by Gs-coupled metabotropic receptors that require A_2A_R or 5-HT_7_ receptor activation [151,152,153]. When pLTF is elicited via the adenosine-dependent mechanism, it is independent of 5-HT receptor activation [146]. The Gs protein-coupled adenosine A_2A_R (GsPCRs) activation induces a downstream signaling cascade that requires exchange protein activated by cAMP (EPAC), protein kinase b (pAkt) signaling via phosphatidylinositol 3-kinases (PI3K) and new protein synthesis of immature tropomyosin-related kinase B isoform rather than BDNF [152,153,154].

It was initially thought that the Q pathway and the S pathway would work together to elicit pLTF. However, it is now known that the serotonin and adenosine-dependent pathways interact via crosstalk inhibition dependent on the severity of AIH. The Q pathway predominantly follows mAIH, and the S-pathway follows sAIH [155]. When a shift from mAIH to sAIH occurs, serotonin shifts to adenosine-dependent pLTF, with greater ATP release and extracellular adenosine accumulation contributing to the shift during severe hypoxic episodes [156,157,158]. The longer the cumulative duration of hypoxia, the greater the accumulation of extracellular adenosine [159]. During mAIH induced pLTF, the S pathway diminishes the Q pathway activity by concurrent, subthreshold activation of spinal A_2A_Rs [133]. When these mechanisms are activated equally, they can cancel each other out and block phrenic motor plasticity, which has profound implications for therapeutic AIH (tAIH) used to treat severe neuromuscular disorders that compromise breathing [159,160,161].

The rationale for treating various neuromuscular disorders with tAIH, a non-invasive treatment modality that consists of brief periods of hypoxic gas mixtures interspersed by periods of normoxia, was initially studied in human [162,163] and intact rat models [164]. These rodent studies showed that daily tAIH treatments activated carotid body chemoreceptors that are required for serotonin-dependent pMF [137,143,155,159,165]. Stimulation of episodic serotonin release then initiated a cell-signaling cascade with the synthesis of BDNF and activation of TrkB, leading to increased synaptic input and motor output of respiratory and motor nuclei, giving rise to pMF [129,145,164,166].

With spinal cord injuries, the disruption between brain and spinal cord pathways results in impaired motor control, breathing control and loss of function below the area of injury. However, around 95% of spinal cord injuries are incomplete (iSCI) [167,168]. The incomplete nature of these injuries leaves spared neural pathways with spinal plasticity that can partially restore recovery of limb function, although the recovery is limited [169,170]. However, because of the limitations with spontaneous plasticity in iSCI patients, there is a need for strategies to further promote spinal plasticity and increase functional recovery [162,163,171].

In animal models, rats with cervical spinal hemisections showed restored breathing compacity following repetitive AIH treatments [135,137,164,172]. The recovery of respiratory function occurred through strengthening the phrenic motor output through the serotonin-dependent S-pathway [129,164]. In addition, tAIH demonstrated enhanced motor function via increased plasticity in somatic motor nuclei and restored forelimb function [164,173]. In humans with iSCI, several recent studies have shown that tAIH enhanced corticospinal synaptic plasticity and showed improved motor function [162,163,171,174]. Trumbower et al. showed that a single AIH treatment improved ankle strength in patients with chronic iSCI that lasted one hour after treatment [163]. Other studies showed combining repetitive AIH with hand opening practice or gait training enhanced hand and walking function in iSCI patients [162,171].

However, results in the rodent and human studies showed variable responses, indicating that other factors may impact the efficacy of tAIH [162,163]. An anesthetized rodent study by Hoffman et al. demonstrated that spinal A_2A_R activation constrained AIH-induced pLTF. Therefore, respiratory plasticity may be modulated by the S-pathway following tAIH [165]. The model they proposed is that both receptor pathways are activated during AIH. However, serotonin-dependent pathways predominate while cross-talk inhibition from A_2A_R-dependent pathways constrains AIH-induced pLTF [165]. Indeed, A_2A_R inhibition in these anesthetized rats enhanced pLTF [165].

Additionally, a study of unanesthetized rats observed that moderate AIH induced diaphragm (dia) pLTF after chronic, not acute, cervical spinal injuries, and a single dose of the A_2A_R antagonist, istradefylline, enhanced dia-LTF in normal rats, but not chronic (8 weeks) cervical (C2) spinal hemisection (C2HS) [175]. Other key observations in SCI rodent models indicate that 2 weeks post-C2HS dAIH enhanced breathing capacity [172,176,177]. Still, functional recovery is adenosine-dependent, and dAIH induced recovery of breathing capacity was less robust eight weeks post-surgery as there was a shift from serotonin-independent to the serotonin-dependent mechanism when transitioning from acute to chronic SCI [172,176,177]. Increased tAIH efficacy was observed following the administration of istradefylline in these chronic SCI animals [172]. It may be surmised that A_2A_R antagonists increase the therapeutic effects of tAIH by releasing the adenosine constraints and further augment respiratory motor output, making A_2A_R inhibition of clinical interest when treating respiratory insufficiency in spinal cord injury and neurodegenerative diseases, albeit depending on the time post-injury [172,176].

These initial findings involving crosstalk inhibition and enhancing tAIH treatment by combining with A_2A_R inhibition in SCI models are also relevant in ALS. A_2A_Rs are upregulated in patients with ALS, specifically in respiratory motor neurons [45,61,64], and ALS animal models show a major loss of phrenic motor neurons in end-stage disease [151]. Despite up to 80% loss of phrenic motor neurons at this stage in ALS, the nerve activity is only reduced to around 50%. By taking advantage of the remaining neurons and A_2A_R increase in patients with ALS, tAIH combined with A_2A_ antagonists could be an effective treatment option that could further preserve pLTF-enhanced breathing capacity. By preserving independent breathing in ALS patients, enhancing the quality of life and extending life duration is possible. Once independent breathing ability is lost for those with ALS, mechanical ventilators are required, and many patients choose end-of-life options.

Several animal studies using a transgenic ALS rodent model (SOD1^G93A^) have provided preliminary data to support this theory. At the end-stage of disease in SOD1^G93A^ rats, a single dose of AIH was restored and showed sustained increase in phrenic nerve burst amplitude via pLTF [151,178,179]. AIH-induced pMF analyzed in young, pre-symptomatic and end-stage SOD1^G93A^ rats showed that the phrenic burst activity was restored to around 50% of normal levels in end-stage and pMF was doubled compared to pre-symptomatic and wild-type rats [178]. End-stage SOD1^G93A^ and wild-type littermates demonstrated that AIH enhanced pLTF occurs via the Q pathway. The serotonin-induced ERK/MAP kinase pathway activation and BDNF protein synthesis were increased in the spared phrenic motor neurons, consistent with Q-pathway requirements for pLTF induction [179]. A_2A_Rs can also activate several intracellular cascades, including downstream signaling via ERK and MAP kinases. After neurotoxin insult, it has been observed that A_2A_Rs are upregulated prior to phrenic motor neuron death (Table 3). The A_2A_ antagonist istradefylline reduced phrenic motor neuron death and preserved diaphragm EMG activity after toxic insults [78]. It also reduced the p38 MAP kinase phosphorylation seen after toxic insult, similar to the observed increase in phosphor-ERK in ALS rodent models that improved phrenic motor neuron survival and diaphragm function [78,166,180].

Based on the discoveries in rodent ALS models, a clinical trial (NCT03645031) is currently recruiting patients with ALS to investigate the effects of a single acute AIH session on respiratory and non-respiratory motor function and EMG (electromyography) activity on patients with ALS and healthy controls [181]. Further investigations are needed to confirm the mechanisms underlying phrenic motor plasticity in ALS to guide new treatment combinations for improved breathing capabilities, which would potentially lead to increased quality of life. These include preliminary human studies in ALS combining A_2A_ antagonists with tAIH treatment that could have significant implications for independent breathing and extended duration of life.

## 8. Conclusions and Future Perspectives

ALS is a complex, multifactorial disease where the activity of adenosine at A_2A_Rs has been shown to play a role in pathogenesis and disease progression. Increased A_2A_R expression has been observed in the spinal cord in both animal models of ALS (e.g., SOD1^G93A^ mice) and in post-mortem tissue from human patients. While dependent on disease stage, this increased expression in the spinal cord is accompanied by enhanced expression and signaling in non-motor areas of the brain as well. These findings suggest that alterations in A_2A_R expression may contribute to progression of the disease. By contrast, relatively little is known about the role of A_1,_ A_2B_ and A_3_ receptors in ALS. Given the known neuroprotective effects of A_1_ receptors [182,183], this can be considered surprising, and this avenue of research merits further attention [63].

Interest in A_2A_Rs as a therapeutic target for ALS has grown exponentially in recent years. Both agonists and antagonists to this receptor have been investigated in animal models of ALS and seem to point to a potential role for pharmacological manipulation of A_2A_ (e.g., in the regulation of phrenic motor facilitation) in the treatment of patients with ALS. However, the timing by which the alterations of the adenosinergic system occur during ALS pathogenesis in patients and animal models is a key factor to completely understand its contribution to disease progression and to identify the proper therapeutic window for putative treatments. Ongoing studies in human patients may help to identify these benefits and may potentially improve the lives of this patient population for which disease-modifying options are limited and treatment usually focuses on symptomatic management.

## Figures and Tables

**Table 2:** Alteration of adenosine and adenosine receptors in ALS and models.

Human	Sample/Model	Tissue/Sample Examined	Finding	Reference
A_2A_R	Postmortem samples from human patients with ALS	Spinal cord	Upregulation of A_2A_R	[64]
A_2A_R	Patients with ALS	Lymphocytes	Upregulation of A_2A_R	[68]
Adenosine	Patients with ALS	CSF	Increase of adenosine level	[61]
ADK	Patients with ALS	Reactive astrocyte/Spinal cord	Upregulation of ADK	[69]
A_1_R	Postmortem samples from human patients with ALS	Spinal cord	No significant change of A_1_R	[64]
**Mouse**				
A_2A_R	SOD1^G93A^ mice	Spinal cord	Upregulation of A_2A_R (early symptomatic stage)	[64]
A_2A_R	SOD1^G93A^ mice	Spinal cord	Decrease of A_2A_R (end stage)	[65]
A_2A_R	SOD1^G93A^ mice	Hippocampus	Increased adenosine A_2A_R levels in hippocampus (pre-symptomatic and symptomatic stage). Impairment of LTP and NMDA receptor function.	[66]
A_1_R	SOD1^G93A^ mice	Spinal cord	No significant change of A_1_R (symptomatic onset period: P100–110)	[64]

**Table 3:** Pharmacological studies of adenosine A_2A_ receptor agonists and antagonists in ALS models (in vitro, in vivo).

	Experimental Model	Cell/Brain Area	Compound	Findings	Reference
***In vitro***					
A_2A_R antagonist	Embryonic SD rat spinal cord cultures	Motor neurons	Istradefylline (1 μM)	Istradefylline protected against kainate-induced motor neuron death	[70]
A_2A_R antagonist, A_2A_R +/−	SOD1^G93A^+ astrocyte induced cell death	Embryonic stem cell-derived motor neuron (ESMN)	Istradefylline (1, 10 μM) A_2A_R +/−	Pharmacological inhibition (istradefylline) and partial genetic ablation of A_2A_R (A_2A_R +/−) significantly protected ESMN from SOD^G93A^+ astrocyte-induced cell death	[71]
A_2A_R agonist, antagonist	Motor neuron cell line	NSC34 cells	Agonist: JMF1907 (30 μM) Antagonist: SCH58261 (10 μM)	JMF1907 enhanced the activity of adenylyl cyclase (AC) and suppressed the aberrant AMPK activity induced by AICAR, the AMPK-triggered mislocalization of TDP-43. These effects of JMF1907 were blocked using an A_2A_R-selective antagonist (SCH58261)	[72]
ADA	C9orf72 or sporadic ALS patients derived induced astrocyte	Astrocyte		RNA and protein levels of ADA were reduced in C9orf72 and sporadic ALS patient cell models. C9orf72 and sporadic ALS induced astrocytes were more susceptible to adenosine-mediated toxicity	[62]
D_2_R agonist, A_2A_R agonist	Motor neuron	Cell line: NSC34 cells	A_2A_R agonist: T1–11 (30 μM) D_2_R agonist: quinpirole (1 μM)	Activation of D_2_R (quinpirole) negatively regulated A_2A_R-evoked cAMP signaling, without significantly affecting the binding affinity of T1–11 toward A_2A_R in NSC34 cells Activation of D_2_R suppressed A_2A_R-mediated protection of TDP-43 mislocalization in NSC34 cells	[73]
***In vivo***					
A_2A_R antagonist	SOD1^G93A^ mice	Spinal cord	Istradefylline (3 mg/kg, ip) starting at P90–95 by daily ip injection (before symptomatic onset period). Disease onset: 121 ± 1.7 day	Istradefylline significantly delayed disease progression	[64]
A_2A_R antagonist	SOD1^G93A^ mice	Hippocampus	Istradefylline (3 mg/kg/day) via drinking water (7.5 μg/mL) starting from 11 weeks to 16–18 weeks (symptomatic) old (early symptomatic disease stage)	Istradefylline rescued LTP impairment and A_2A_R levels	[66]
A_2A_R agonist	SOD1^G93A^ mice	Spinal cord	CGS21680 (5 mg/kg/day, ip): Starting at 8 weeks of age (before the clinical manifestation of the disease)	CGS21680 treatment slowed the onset of motor neuron degeneration (12 weeks) and muscle weakness	[74]
A_2A_R agonist	SOD1^G93A^ mice (Electrophysiological recordings)	Neuromuscular junction	CGS21680 (5 nM) pre-symptomatic mice (4–6 weeks) symptomatic mice (12–14 weeks)	In pre-symptomatic mice (4–6 weeks) the excitatory A_2A_R-mediated effects on neuromuscular transmission are exacerbated In symptomatic mice (12–14 weeks) the excitatory A_2A_R-mediated effects on neuromuscular transmission were absent	[75]
A_2A_R agonist	TDP-43 transgenic mice	Spinal cord	JMF1907 (111 mg/ mouse/day, sc) using ALZET osmotic minipump. Starting from 6 weeks to 23 weeks old (from presymptomatic)	JMF1907 markedly reduced the activation of AMPK JMF1907 also improved motor function based on rotarod performance and forelimb grip strength	[72]
A_2A_R agonist, antagonist, A_1_R antagonist	SOD1^G93A^ mice	Motor performance, survival	A_2A_R agonist: CGS21680 (2.5 mg/kg, ip): five times per week. A_2A_R antagonist: istradefylline (3 mg/kg/day) via drinking water (0.25 mg/mL). A_1_R antagonist: DPCPX (0.75 mg/kg, ip): five times per week. Starting from 70 days of age (presymptomatic stage)	Neither the stimulation nor the blockade of adenosine A_2A_R modified the progressive loss of motor skills or survival of SOD^G93A^ mice. Blockade of adenosine A_1_R from the presymptomatic stage significantly attenuated motor disease progression and induced a non-significant increase of median survival in ALS mice.	[76]
A_1_/A_2A_R antagonist	SOD1^G93A^ mice	Spinal cord	Caffeine (0.3 mg/mL) via drinking water. Starting from 70 days of age (before the onset of symptoms)	Caffeine intake significantly shortened the survival of SOD^G93A^ mice	[65]
D_2_R agonist, A_2A_R agonist	A315T TDP-43 transgenic mice	Spinal cord, motor performance (grip strength)	A_2A_R agonist: T1–11 (0.25 mg/mL) via drinking water. D_2_R agonist: Quinpirole (6 mg/kg, ip/day). Starting from 7 to 10 weeks old	Activation of D_2_R inhibited the A_2A_R -mediated beneficial effects (rescuing effect of T1–11 on TDP-43 mislocalization and impaired grip strength)	[73]
A_1_R agonist, antagonist, A_2A_R agonist, antagonist	SOD1^G93A^ mice	Neuromuscular junction	A_1_R agonist: CPA (50 nM) A_1_R antagonist: DPCPX (50 nM) A_2A_R agonist: CGS21680 (5 nM) A_2A_R antagonist: SCH58261 (50 nM) pre-symptomatic mice (4–6 weeks) symptomatic mice (12–14 weeks)	In pre-symptomatic mice (4–6 weeks), there is a loss of A_1_R-A_2A_R functional crosstalk. In symptomatic mice (12–14 weeks), there is increased A_1_R tonic activation	[77]
***In vivo* (phrenic motor neurons)**					
A_2A_R antagonist	Intrapleural CtB-Saporin injected rats (neurotoxic model of respiratory motor neuron death)	phrenic motor neuron	Istradefylline twice daily, for a total dose of 1 mg/kg/day	Increased A_2A_R expression following CtB-Saporin injections. Istradefylline reduced phrenic motor neuron death and preserved diaphragm EMG activity	[78]
